# Deciphering the intricate linkage between the gut microbiota and Alzheimer's disease: Elucidating mechanistic pathways promising therapeutic strategies

**DOI:** 10.1111/cns.14704

**Published:** 2024-04-07

**Authors:** Junyi Liang, Yueyang Wang, Bin Liu, Xiaohong Dong, Wenhui Cai, Ning Zhang, Hong Zhang

**Affiliations:** ^1^ Heilongjiang University of Traditional Chinese Medicine Harbin Heilongjiang Province China; ^2^ Jiamusi College Heilongjiang University of Traditional Chinese Medicine Jiamusi Heilongjiang Province China; ^3^ Heilongjiang Jiamusi Central Hospital Jiamusi Heilongjiang Province China

**Keywords:** Alzheimer's disease, gut microbiota, gut–brain axis, mechanisms, treatment

## Abstract

**Background:**

The gut microbiome is composed of various microorganisms such as bacteria, fungi, and protozoa, and constitutes an important part of the human gut. Its composition is closely related to human health and disease. Alzheimer's disease (AD) is a neurodegenerative disease whose underlying mechanism has not been fully elucidated. Recent research has shown that there are significant differences in the gut microbiota between AD patients and healthy individuals. Changes in the composition of gut microbiota may lead to the development of harmful factors associated with AD. In addition, the gut microbiota may play a role in the development and progression of AD through the gut–brain axis. However, the exact nature of this relationship has not been fully understood.

**Aims:**

This review will elucidate the types and functions of gut microbiota and their relationship with AD and explore in depth the potential mechanisms of gut microbiota in the occurrence of AD and the prospects for treatment strategies.

**Methods:**

Reviewed literature from PubMed and Web of Science using key terminologies related to AD and the gut microbiome.

**Results:**

Research indicates that the gut microbiota can directly or indirectly influence the occurrence and progression of AD through metabolites, endotoxins, and the vagus nerve.

**Discussion:**

This review discusses the future challenges and research directions regarding the gut microbiota in AD.

**Conclusion:**

While many unresolved issues remain regarding the gut microbiota and AD, the feasibility and immense potential of treating AD by modulating the gut microbiota are evident.

## Graphical abstract

This review delves into the intricate relationship between the gut microbiota and Alzheimer's disease (AD), unraveling the close association between microbial changes and disease mechanisms. By elucidating relevant pathways, we present promising therapeutic strategies involving the modulation of the gut microbiota, opening new avenues for innovative treatments in AD. Graphical Abstract created By Figdraw.

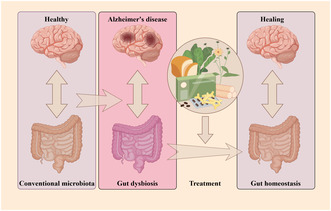

## 1 INTRODUCTION

The gut microbiota constitutes a complex and diverse microbial consortium within the human alimentary canal, numbering approximately 10^14^, encompassing bacteria, fungi, viruses, archaea, and protozoa. Its abundance and diversity are subject to the modulation by various factors.^1^ The intestinal microbiota plays a pivotal role in training the immune system, facilitating food digestion, regulating endocrine and neural signal transduction, pharmaceutical metabolism, detoxification, among other significant functions.^2^

Alzheimer's disease (AD), as the most prevalent neurodegenerative disorder among the global elderly population, manifests neuropathological features encompassing tau protein entanglements, β‐amyloid (Aβ) plaques, as well as the loss and impairment of neurons and synapses. Individuals afflicted with AD commonly exhibit apathy and depression, concomitant with impairments in communication, judgment, and cognition.^3^ Epidemiological reports over the past two decades delineate a marked upward trajectory in the incidence, prevalence, and mortality rates of AD, affecting millions worldwide.^4^, ^5^ In recent years, heightened attention has been directed toward the role of the gut microbiota in AD. Imbalances in the gut microbiota have been implicated in central nervous system (CNS) aberrations.^6^ The gut microbiota, through the gut–brain axis, exerts influence on the brain involving immune, metabolic, endocrine, and neural modalities.^7^ Although pivotal in the pathogenesis of AD, the intricate mechanisms underlying the involvement of the gut microbiota remain elusive. The composition of the gut microbiota emerges as a potential therapeutic target for addressing inflammation and metabolic dysregulation. This review aims to elucidate the interplay between the gut microbiota and AD, explore its potential utility as a novel diagnostic tool, and investigate the feasibility of various intervention measures as adjunctive therapeutic strategies to alleviate disease progression.

## 2 CLASSIFICATION AND FUNCTIONS OF THE GUT MICROBIOTA

The gut microbiota is a complex and dynamic ecosystem housing numerous microorganisms, with colonic bacteria being the most abundant and diverse. Currently, the predominant phyla in the intestinal microbial community are *Firmicutes*,^8^, ^9^, ^10^ *Bacteroidetes*,^11^, ^12^, ^13^ *Actinobacteria* ^14^, ^15^, ^16^ and *Proteobacteria*.^17^, ^18^, ^19^ *Firmicutes* and *Bacteroidetes* together make up about 90% of the gut microbiota (Table 1). The ecological roles, functional contributions, and interplay among these bacterial phyla remain under investigation, constituting a vibrant realm of ongoing scholarly inquiry.

**TABLE 1:** Taxonomy and functions of the gut microbiota.

Category	Main microbiota	Effect	References
Firmicutes	Clostridium coccoides group; Butyrivibrio; Clostridium; Coprococcus; Dorea; Eubacterium; Lachnospira; Roseburia; Ruminococcus; Lactobacillus	Engaging in the synthesis of short‐chain fatty acids; fermenting carbohydrates to produce lactic acid, aiding in digestion and absorption; acidifying the intestinal environment, impeding the adherence of harmful bacteria to the intestinal epithelium; stimulating the production of immunoglobulins, bolstering host immunity	8, 9, 10
Bacteroidetes	Bacteroides; Prevotella; Porphyromonas	Engaging in carbohydrate fermentation, polysaccharide metabolism, bile acid and steroid metabolism, and maintaining intestinal homeostasis are among the involved processes; pathogenic pseudomonads can induce abdominal abscesses, wound infections, and bacteremia	11, 12, 13
Proteobacteria	Enterobacteraceae; Vibrionaceae; Pseudomonadaceae	Exerting cytotoxic effects on intestinal epithelial cells; hindering the oxidation of butyrate salts, thereby leading to disruption of intestinal barrier function	17, 18, 19
Actinobacteria	Bifidobacterium	Acidifying the intestinal environment inhibits the proliferation of putrefactive and pathogenic bacteria, fosters the production of vitamins and amino acids, stimulates immune responses, mitigates inflammatory reactions, and safeguards the intestinal barrier, thereby reducing the translocation of endotoxins into the bloodstream	14, 15, 16

The intestinal microbiota, with its intricate mechanisms, profoundly shapes human physiological processes, delving into realms such as immunity, metabolism, and the nervous system, thereby holding paramount significance in the evolution of health and disease. Short‐chain fatty acids (SCFAs), generated through the fermentation of dietary fiber by the intestinal microbiota, play a pivotal role in modulating immune system function.^20^, ^21^, ^22^, ^23^ Moreover, metabolic byproducts produced by the intestinal microbiota, such as purines, tryptophan, and lipids, exert expansive effects on human physiological functions.^20^, ^24^ Tryptophan metabolism, via the kynurenine pathway, gives rise to downstream products that control biological processes such as neural transmission, inflammation, and immune responses.^25^, ^26^ The intimate connection between the intestinal microbiota and lipid metabolism intertwines and reciprocally influences each other.^27^ The composition and abundance of lipids not only impact the microbiota but also, in turn, the microbiota, through the production of short‐chain fatty acids and metabolites like trimethylamine, partake in bile acid metabolism, significantly influencing host lipid metabolic pathways.^28^, ^29^ These interactions notably affect lipid levels in the blood and tissues, closely correlating with the onset of metabolic diseases.^30^ Finally, the intestinal microbiota, by regulating metabolites associated with Proteobacteria, such as taurine, histamine, and spermine, harmonizes the regulation of inflammasomes, profoundly influencing the host's susceptibility to diseases.^31^, ^32^

## 3 ALZHEIMER'S DISEASE

Currently, a comprehensive consensus prevails concerning the pathogenic mechanisms underpinning AD, encompassing myriad pivotal facets.^3^ Chief among these considerations is the amyloid cascade hypothesis, postulating that the anomalous aggregation of Aβ assumes a central role in the pathological progression.^33^, ^34^ Subsequently, the prominence of inflammatory responses in the onset of AD is acknowledged, wherein instigated inflammatory processes may evoke neuronal damage.^35^, ^36^ Furthermore, factors spanning glucose metabolism aberrations, compromise of the BBB, anomalies in tau protein, disturbances in autophagic‐lysosomal pathways, mitochondrial functionality, cholinergic transmission, oxidative stress, and genetic susceptibility, among others, are posited to exert influence upon the progression of AD.^3^ Recent inquiries suggest that dysbiosis within the gastrointestinal microbiota may emerge as a pivotal factor in the pathogenesis of AD, impacting the deposition of Aβ and the occurrence of neuroinflammation.^37^ These myriad factors intricately intertwine, collectively propelling the progression of AD.

## 4 THE GUT MICROBIOTA AND AD

**FIGURE 1:**
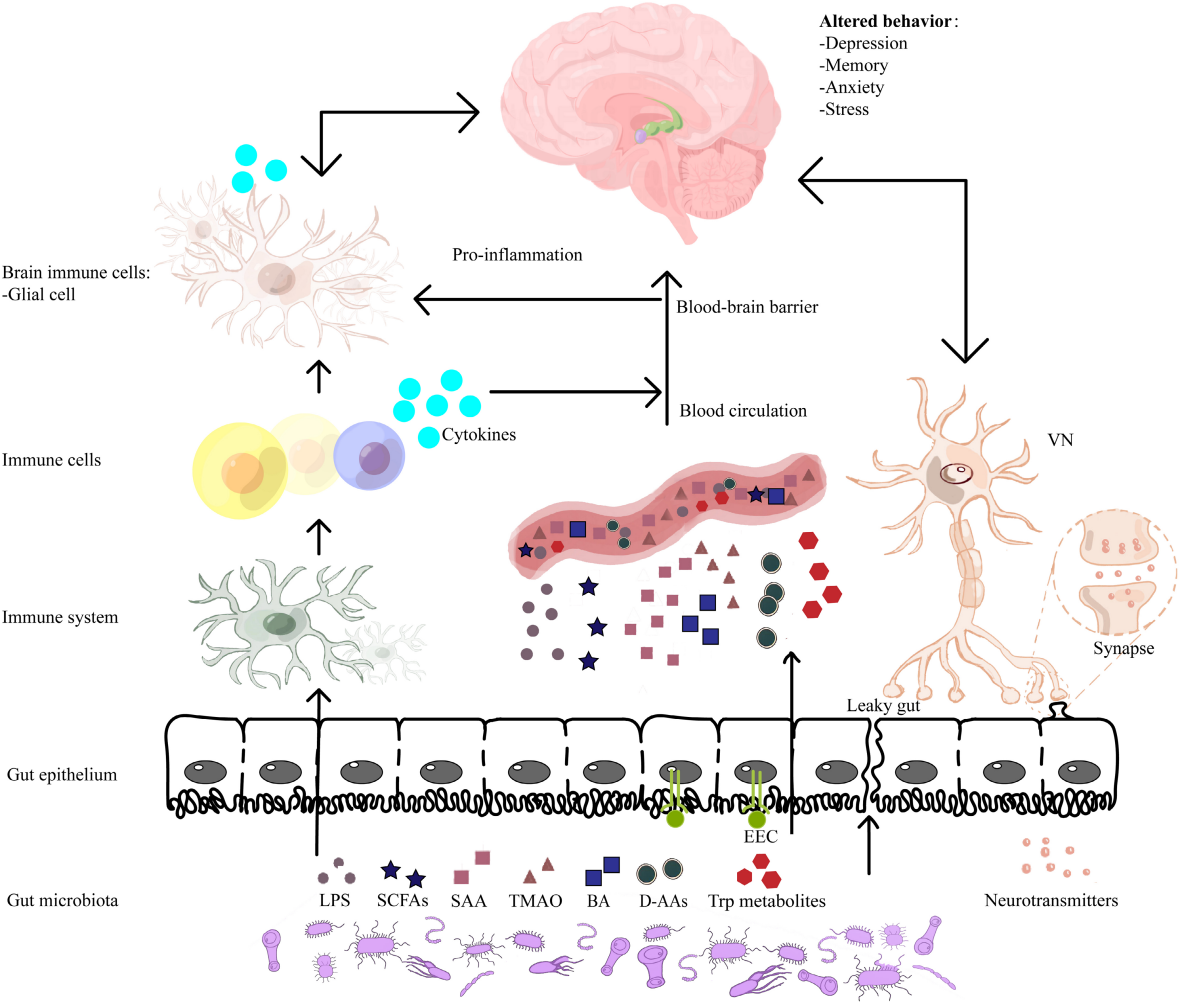
Communication between the gut microbiome and brain. A pictorial representation elucidating the manifold established bidirectional conduits of communication within the gastrointestinal‐neuronal axis in relation to the gut microbiota associated with AD, encompassing: (1) pathways of immune modulation, (2) transmission of signals pertaining to gut endocrine secretions and microbial metabolites, and (3) the neural pathway of the VN. BA, bile acids; D‐AAs, D‐amino acids; EEC, enteroendocrine cell; LPS, lipopolysaccharide; SAA, serum amyloid A; SCFAs, short‐chain fatty acids; TMAO, trimethylamine‐N‐oxide; Trp, tryptophan; VN, vagus nerve.

The gut–brain axis is a complex signaling pathway that establishes bidirectional communication between the gastrointestinal tract and the CNS, allowing for the integration of peripheral mechanisms with cognitive centers in the brain.^38^ The gut microbiota, through both direct and indirect chemical signaling pathways, plays a pivotal role in regulating neuronal plasticity, epigenetics, gene expression, as well as the synthesis and release of neurotransmitters. Furthermore, the microbial byproducts released by the gut microbiota critically modulate signaling pathways associated with the regulation of pro‐inflammatory cytokine production. These pathways, encompassing metabolism, endocrine, neural, and immune pathways, act independently or synergistically, intricately intertwined with the pathogenesis of AD (Figure 1, Table 2).

**TABLE 2:** Involvement of the gut microbiota in regulating AD pathology.

Category	Regulator	Associated microbes	Route	Effect	References
Metabolites of microorganisms	GABA	Bifidobacterium; Lactobacillus	Neuronal	The disruption of synaptic excitatory and inhibitory balance leads to the facilitation of pathological Aβ propagation. Excessive activation of NMDARs triggers calcium‐dependent intracellular signaling pathways, compromising energy metabolism, generating highly reactive free radicals, inducing oxidative stress, and ultimately culminating in cellular demise	59, 60, 61
	SCFAs	Bacteroidetes; Firmicutes	Systemic; immune; Neuronal	Modulate the permeability of the BBB; foster the maturation and functionality of microglial cells; govern the levels of pro‐inflammatory cytokines; and promote as well as regulate the activity of T cells	22, 23, 43, 89, 93
	Tryptophan metabolites	Bifidobacterium; Bacillus	Neuronal; Systemic	Modulating the activity of astrocytes; regulating the levels of neurotransmitters, inflammation, and immune response	24, 26, 50, 51, 53
	Bile acids	Bifidobacterium; Clostridium; Lactobacillus; Bacteroides	Systemic	Regulating the permeability of the BBB plays a pivotal role. Modulating lipid metabolism exerts influence on Aβ levels within the brain	29, 83, 84, 85
	D‐AAs	Enterococci; Lactobacillus	Systemic; Neuronal	Alleviating cellular apoptosis demonstrates neuroprotective outcomes; influencing the proliferation of neural stem cells and neuronal differentiation; modulating NMDAR activity; influencing synaptic plasticity, neural ontogenesis, as well as the processes to learning and memory	63, 64, 72, 73, 74, 75, 76, 77
	TMAO	Lachnoclostridium; Clostridium	Systemic	Harnessing the potential of microglial cells to exert influence over neuroinflammation; inducing neuronal senescence; Attenuating the expression levels of synaptic plasticity‐associated proteins; compromising the integrity of mitochondria	86, 87, 88
The intestinal microbiota and endotoxins	LPS	Bacteroides fragilis	Immune; Systemic	Activation of NF‐κB (p50/p65), provocation of the innate immune system, impairment of the IEB, and disruption of the BBB	95, 98, 99, 100, 101, 102
	SAA	Bifidobacterium; Lactobacillus; Salmonella; *E. coli*	Immune; Systemic	Facilitating neuroinflammation through the mediation of Th17 cells, modulating cholesterol metabolism, and activating glial cells by means of PI3K activation in microglia and astrocytes	103, 104, 105, 106, 107
The gut microbiota and the nervous system	Vagus nerve	/	Neuronal	Modulating the expression of glutamate receptors; augmenting synaptic plasticity; and engaging in the synthesis and release of acetylcholine	112, 113, 114

Abbreviations: Aβ, amyloid beta; BBB, blood–brain barrier; D‐AAs, D‐amino acids; GABA, γ‐aminobutyric acid; IEB, intestinal epithelial barrier; LPS, lipopolysaccharides; NMDAR, *N*‐methyl‐d‐aspartate receptors; SAA, Serum amyloid A; SCFAs, short‐chain fatty acids; TMAO, trimethylamine N‐oxide.

### 4.1 Intestinal microbiota alterations in AD

The present inquiry elucidates significant modifications in the gut microbiota of individuals afflicted with AD and their corresponding animal models.^6^ The findings signify an augmentation in specific microbial phyla within the gastrointestinal microbiota of AD patients, a phenomenon concurrent with amyloid deposition in the cerebral cortex of cognitively impaired individuals.^39^, ^40^ Bacterial composition in the fecal matter of AD patients demonstrates a positive correlation with amyloid levels, a decrease in *lactobacilli*, and an increase in lipopolysaccharides (LPS) and *Escherichia coli*.^41^, ^42^ Investigations involving germ‐free and AD animal models collectively posit an interrelation between gut microbiota and Aβ pathology.^43^, ^44^, ^45^, ^46^ These investigations elucidate the intricate alterations in the gut microbiota during the pathogenesis of AD.

### 4.2 The gut microbiota and metabolism

The microbiota's metabolism may regulate neurotransmitter or their precursor biosynthesis, thereby influencing the gut–brain axis of the microbiota via neuroendocrine pathways.

The tryptophan‐related metabolites orchestrated by the gut microbiota may exert a discernible influence on AD. Markedly diminished levels of indole and tryptophan (Trp) were ascertained in the plasma and erythrocytes of AD patients, in comparison to the control group.^47^, ^48^ Tryptophan derivatives, secreted by the gut microbiota, intricately modulate astrocytes and microglia through the aryl hydrocarbon receptor (AhR) signaling pathway.^49^ Recent investigations have unveiled that indole metabolites extracted from the gut microbiota upregulate AhR, suppress the activation of the NF‐κB pathway, alleviate the formation of NLRP3 inflammasomes, thereby diminishing the release of inflammatory cytokines, ultimately mitigating neuroinflammation in APP/PS1 mice.^50^ Consequently, the modulation of astrocyte activation, potentially alleviating central nervous system inflammation, may be facilitated by the supplementation of indole or Trp metabolites, such as those generated by bacterial tryptophanases.^51^ 5‐hydroxytryptamine (5‐HT), a pivotal neurotransmitter, undergoes regulation in its metabolism through the modulation of tryptophan hydroxylase in neurons and intestinal mucosal cells.^52^, ^53^ The impact of 5‐HT extends to monocytes and macrophages, thereby orchestrating inflammatory responses. This immunomodulation may be interconnected with neuroinflammation.^54^ Investigations reveal that 5‐HT, via activation of the 5HT2AR/cAMP/PKA/CREB/Sirt1 pathway and the NF‐κB pathway, modulates the transcription of Toll‐like receptor 2 (TLR2) and TLR4, thereby eliciting phagocytic responses in microglial cells to stimuli.^55^, ^56^, ^57^ Simultaneously, it regulates the release of inflammatory cytokines such as TNF‐α, IFN‐γ, IL‐1β, IL‐17, and IL‐6,^58^ ultimately influencing neuroinflammation. Additionally, the gut microbiota, including *Bifidobacteria* and *Lactobacilli*, metabolize glutamate to produce γ‐aminobutyric acid (GABA), the primary inhibitory neurotransmitter in the CNS.^59^ Disruptions in glutamate neurotransmission, such as impaired GABA signaling, decreased glutamate concentrations, and downregulation of glutamate transporters, may contribute to cognitive impairment in AD.^60^, ^61^

The reciprocal interplay of enteric microbiota begets an array of liberated D‐amino acids (D‐AAs), encompassing D‐serine, D‐glutamate, and D‐aspartate.^62^, ^63^, ^64^ These D‐AAs, disseminated via the bloodstream, subsequently modulate the neurotransmitter system within the cerebral milieu.^65^, ^66^ Inquiries divulge attenuated plasma levels of D‐glutamate in individuals with MCI and AD in comparison to the control cohort, with conspicuous deviations in serum D‐serine levels as well.^67^, ^68^, ^69^ These modifications may manifest disparately across sundry stages of AD.^70^, ^71^ D‐AAs may exert an impact on AD through various mechanisms. D‐serine has the capacity to attenuate neuronal demise, forestall cellular apoptosis,^72^ stimulate neural stem cell proliferation, and neuronal differentiation,^73^ modulate the activity of *N*‐methyl‐d‐aspartate receptors, thereby influencing synaptic plasticity and neurodevelopment.^74^, ^75^ Furthermore, D‐serine, by inhibiting the JNK signaling pathway, ameliorates cognitive impairment induced by hippocampal Aβ42 injection.^76^ Investigations also indicate that dietary or aqueous supplementation of l‐Serine or d‐Serine alleviates memory deficits associated with reduced D‐Serine levels and plasticity defects in 3xTg‐AD mice.^77^ Additionally, the diminished levels of D‐Glutamate in AD warrant further exploration of its neurophysiological implications.^78^, ^79^ Future research should delve into exploring the impact of D‐AAs in the gut microbiota on host health and disease, particularly regarding the specific mechanistic roles of D‐AAs in AD.

The gut microbiome also plays a crucial role in the metabolism and bioconversion of bile acids (BA). Experimental and clinical data have shown that there are disruptions in BA signaling in AD brain samples compared to cognitively normal individuals, as well as decreased concentrations of BA and abnormal cholesterol metabolism in serum, suggesting that the microbiome may impact AD through its effect on BA.^80^, ^81^, ^82^, ^83^ BA abnormalities may also increase BBB permeability when rats undergo bile duct ligation or receive injections of deoxycholic acid.^84^ Interestingly, endogenous hydrophilic BA Tauroursodeoxycholic acid (TUDCA) was found to reduce brain Aβ levels by regulating lipid metabolism after feeding AD mice a diet containing TUDCA for six months.^85^

The synthesis of trimethylamine *N*‐oxide (TMAO) by the intestinal microbiota exhibits an inverse correlation with cognitive function in the elderly.^86^ Possessing the capacity to traverse the BBB, TMAO augments the activation of microglial cells and the release of inflammatory mediators.^87^ Investigations involving the administration of exogenous TMAO to SAMR1 and SAMP8 mice reveal its potential to induce senescence in the CA3 region neurons of the hippocampus.^88^ Furthermore, TMAO, by inhibiting the mTOR signaling pathway, exacerbates synaptic damage, diminishes the expression levels of proteins associated with synaptic plasticity, thereby compromising mitochondrial integrity.^88^ These findings underscore the pivotal role TMAO may play in the induction of AD.

SCFAs constitute metabolic byproducts emanating from the microbial metabolism of dietary fiber within the gastrointestinal milieu. Their functional purview transcends the mere modulation of the enteric nervous system.^89^ Monocarboxylic acid transporters on endothelial cells enable the passage of SCFAs across the BBB.^90^ In the genesis and progression of AD, SCFAs exert a pivotal regulatory influence, impacting the functionality of microglial cells and thereby modulating the course of AD. Investigations reveal that, through interaction with the GPR43 receptor, SCFAs can ameliorate deficiencies in both form and function within the microglial cells of germ‐free murine brains.^43^ In vitro experiments demonstrate the capability of SCFAs to attenuate the activity of histone deacetylases and nuclear translocation of NF‐κB, thereby directly regulating and significantly diminishing LPS‐induced microglia activation while modulating neuroinflammatory responses.^91^ Furthermore, SCFAs can influence the BBB. Colonizing germ‐free mice with butyrate‐producing *Clostridium* or propionate‐producing *Bacteroides*, as well as administering oral sodium butyrate, reduces BBB permeability and increases occludin protein expression in the frontal cortex and hypothalamus.^92^, ^93^ Intraperitoneal injection of sodium butyrate improved neurological functional deficits and restored BBB permeability in a brain injury rat model.^94^ Further investigation is warranted to ascertain whether SCFAs exert analogous effects on other cells within the cerebral milieu.

### 4.3 The gut microbiota and endotoxins

The gut microbiota may modulate signaling pathways through the generation of LPS, amyloid‐like proteins, and related signaling molecules, thereby influencing the pathogenesis of AD. The presence of bacterial‐derived LPS in the brains of AD patients has been substantiated.^95^ Various factors, including aging, vascular defects, and diseases, contribute to the disruption of the BBB. Additionally, dysbiosis of the intestinal microbiota, leading to the abnormal production of bacterial‐derived LPS and amyloid‐like proteins, compromises the integrity of the intestinal barrier. This disruption amplifies the levels of cytokines associated with AD, facilitating the “leaky” of neurotoxic molecules to the cerebrovascular system.^96^ Consequently, this cascade triggers neuroinflammation and neuronal damage.^97^ Moreover, this inflammatory response may precipitate structural remodeling of the BBB and heightened permeability, ultimately fostering the genesis of AD. The entire cascade involves an augmentation in reactive oxygen species levels, initiation of the NF‐κB signaling pathway, upregulation of miRNA‐34a, downregulation of TREM2, and compromise of microglial phagocytic function, collectively propelling the accumulation of Aβ. Furthermore, LPS can selectively activate TLR4, leading to the generation of pleiotropic cytokines/chemokines, thereby modulating inflammation, innate, and subsequent adaptive immune responses.^98^, ^99^, ^100^, ^101^, ^102^

Serum amyloid A (SAA) is a significant acute‐phase protein associated with gut microbial ecology and inflammation.^103^ It has been locally detected in the brains of AD patients and co‐localized with senile plaques. SAA can influence AD by promoting Th17 cell‐mediated neuroinflammation, regulating cholesterol metabolism, and activating glial cells. Experimental evidence demonstrates that SAA can directly induce the differentiation of Th17 cells, leading to increased expression of Th17 pro‐inflammatory cytokines, such as IL‐17 and IL‐22, within the hippocampus; these cytokines exhibit elevated concentrations in cerebrospinal fluid and serum.^104^, ^105^ Furthermore, SAA can affect AD by activating microglia and astrocytes via the PI3K pathway.^106^ SAA also affects cholesterol metabolism, which plays a vital role in brain‐derived trophic factors.^107^ Additionally, the gut microbiota can directly stimulate Th17 cells, thereby triggering inflammation.^108^

### 4.4 The gut microbiota and vagus nerve

The intestinal microbiota serves as a pivotal nexus between the microbial community of the gastrointestinal tract and AD through various anatomical pathways, including the vagus nerve (VN), the spinal cord and the hypothalamus‐pituitary–adrenal axis of the neuroendocrine system.^109^

The VN consists of mainly afferent (80%) and efferent (20%) fibers, assumes a pivotal role in the transmission of signals between the brain and the intestinal tract.^109^ The VN is the primary route for gut microbes to influence the brain, affecting metabolic and feeding behaviors, as well as inflammatory responses that link the brain, the gut, and other organs.^110^ Enteroendocrine cells containing glucose‐dependent insulinotropic peptide, glucagon‐like peptide‐1, and peptide YY can directly communicate with VN afferent fibers, which transmit information to the central autonomic network for analysis and integration, including the paraventricular nucleus, locus coeruleus, hypothalamus, and limbic system, such as the thalamus, amygdala, and hippocampus.^109^, ^111^ Studies indicate that chronic VN stimulation in AD rats alters glutamate receptor levels, thereby ameliorating memory.^112^ Additionally, VN stimulation activates the locus coeruleus, resulting in the release of catecholamines in the hippocampus and neocortex, which enhances synaptic plasticity and reduces inflammatory signals.^113^ The VN also serves as a direct conduit for signals from certain bacteria, such as *Paenalcaligenes hominis*, to enter the brain.^114^ Additionally, VN efferent fibers can synthesize and release acetylcholine, influencing not only cholinergic neurons but also exerting anti‐inflammatory effects by binding to α‐7 nicotinic acetylcholine receptors in macrophages, thereby inhibiting TNF‐α secretion.^115^ The interplay of the VN in the triad of the gut microbiota, behavior, and neurodegenerative diseases is intricate. However, clinical evidence conclusively proving its role in neurofunction demands further profound investigation.

## 5 AD TREATMENT STRATEGIES FOR THE MICROBIOTA

Given the association between the gut microbiota and AD, therapeutic interventions targeting the composition of the gut microbiota, encompassing dietary adjustments, probiotics, antibiotics, and pharmacological treatments, hold the potential to alleviate and treat AD through various avenues (Table 3).

**TABLE 3:** Therapeutic intervention of AD through gut microbiota manipulation.

Category	Drug	Effect	References
Dietary ways	Ketogenic diet	Minimizing bifidobacteria abundance; reducing pro‐inflammatory cell levels; improving cerebral vasculature and BBB functionality	117
	Mediterranean diet	Attenuating the risk of AD onset	118
	Fermented food products	Alleviate inflammatory mediators	119
	Dietary fiber	Interfering the formation of soluble neurotoxic Aβ aggregates	120
	Dietaryinulin	Elevating gastrointestinal microbiota metabolites, including SCFAs, tryptophan‐derived metabolites, and bile acids; reducing hippocampal inflammation gene expression.	121
Probiotic	Probiotic supplementation(BGN4 and BORI)	Heighten the serum levels of blood‐BDNF	125
	Bifidobacterium breve strain A1	Diminishing the expression of inflammation and immune response genes in the hippocampus	127
	Probiotic formulation(SLAB51)	Diminishing Aβ aggregation and partially restore compromised neuronal proteins	126
	Probiotic bacterial strain(L. rhamnosus HA‐114)	Demonstrating neuroprotective properties	124
Pharmacy strategy	GV‐971 (sodium oligo‐mannurarate)	Attenuating intestinal dysbiosis; regulating neuroinflammation	128
	Broadspectrum combinatorial antibiotic treatment	Diminishing Aβ plaque deposition; attenuating the local neuroglial cell response to plaques	130
TCM and formulations	Patchouli alcohol	Mitigating intestinal microbiota dysbiosis; suppress the C/EBPβ/AEP signaling pathway in the brain and colon; modulating Aβ levels, tau phosphorylation and neuroinflammation	37, 133
	Scutellaria baicalensis root	Modulating the gut microbiota composition and their metabolic byproducts, lipid; enhancing glucose metabolism; improving cognitive function	134
	Morinda officinalis	Preserving the gut microbiota diversity and stability; enhancing neuronal functionality; mitigating oxidative stress and inflammation	135, 136
	Cyanidin‐3‐O‐glucoside	Promoting the relative abundance of microbial communities; reducing inflammatory biomarkers	137
	Liuwei‐Dihuang Decoction	Modulating gut microbiota composition; improving cognitive impairment	141, 142
	Chaihu‐Shugan‐San	Modulating intestinal inflammation and the microbiota; achieving the induction of NF‐κB‐mediated BDNF expression	143
	Huanglian‐Jiedu Decoction	Regulating inflammation; inhibiting lipid accumulation	140
	Jia‐Jian‐Di‐Huang‐Yin‐Zi Decoction	Repressing the activation of microglial cells and astrocytes; safeguarding the ultrastructure of the BBB and tight junction proteins	144
Fecal microbiota transplantation	Transplanting healthful gut microbiota	Mitigating burden of Aβ plaques; diminishing levels of soluble Aβ40 and Aβ42; changing cognitive deficits	146, 147

Abbreviations: AD, Alzheimer's disease; Aβ, amyloid beta; BBB, blood–brain barrier; SCFAs, short‐chain fatty acids; BDNF, blood–brain‐derived neurotrophic factor.

### 5.1 Diet

By influencing the types and abundance of gut bacteria, specific foods and dietary patterns can sustain host equilibrium. Research conducted on clinical trials and animal models has indicated that ketogenic diets that are high in fat and low in carbohydrates can offer symptomatic relief and improve the course of “AD”.^116^ The ketogenic diet can selectively diminish the population of *Bifidobacterium* in the gut and reduce pro‐inflammatory Th17 cells.^117^ Similarly, high adherence to the Mediterranean diet has been shown to reduce the risk of AD by 41%.^118^ This effect may be attributed to the propensity of the Mediterranean diet to enhance the abundance of bacteria diminished in AD while concurrently diminishing the prevalence of bacteria heightened in AD. Furthermore, alternative dietary patterns such as high‐fermentable foods,^119^ dietary fiber,^120^ and Dietaryinulin^121^ have also been discovered to confer beneficial effects on AD. Nevertheless, further research is required to ascertain how diets and their components impact the microbiota–gut–brain axis and whether the effects of diets on the microbiota translate to changes in overall brain function.

### 5.2 Probiotics

Probiotics, recognized as a category of active microorganisms capable of endowing the host with health benefits, have garnered considerable attention. Their positive impact on cerebral well‐being, particularly in enhancing cognitive abilities, is increasingly evident, notably in the context of AD or MCI, as indicated by the modulation of the microbiota–gut–brain axis.^122^, ^123^, ^124^ Investigations reveal that the administration of BGN4 and BORI probiotics among the elderly elevates serum levels of neurotrophic factors.^125^ Treatment of early‐stage AD mice with the probiotic preparation SLB51 demonstrates not only an influence on intestinal microbial communities and plasma concentrations of metabolites but also a reduction in Aβ aggregation and the restoration of certain impaired neural protein autophagy pathways.^126^ Furthermore, it is highlighted that the *Bifidobacterium A1* strain has the capacity to inhibit hippocampal inflammation and the expression of genes associated with immune responses in AD mice.^127^ Despite the current supportive evidence for the potential therapeutic role of probiotics, the development of an efficacious and safe probiotic formulation for the prevention or treatment of AD necessitates further profound inquiry.

### 5.3 Pharmacological strategies

In recent times, there has been research on pharmacogenomics and the gut microbiome, which has provided a platform for developing novel approaches to treat AD. The modulation of gut microbiota through drug interventions may offer a promising method of treating AD. GV‐971, a low‐molecular‐weight sodium oligo‐mannuronate, can inhibit gut dysbiosis and related accumulation of phenylalanine/isoleucine in AD mouse models, controlling neuroinflammation and reversing cognitive impairment.^128^ Furthermore, antimicrobial agents are frequently employed on an extensive scale for the elimination or prophylaxis of bacterial colonization within the human organism, as opposed to selectively targeting distinct bacterial strains. Consequently, the application of broad‐spectrum antimicrobials may have repercussions on the constitution of the intestinal microbiota. Despite established findings in the AD murine model suggesting that prolonged administration of broad‐spectrum antimicrobials can diminish the deposition of Aβ plaques and mitigate the localized glial cell response to these plaques, contemporary scientific evidence remains inadequate to substantiate the deployment of antimicrobials as a therapeutic modality for AD.^129^ In AD, the impacts of antimicrobials may encompass a comprehensive and potentially paradoxical spectrum, contingent upon the nature of the antimicrobial agent and the specific roles of the microbiota in the pathogenesis of AD.^130^

Traditional Chinese medicine (TCM) formulations have shown advantages over other pharmacological agents, such as their complex composition, multiple target modulation, low side effects and high biocompatibility.^131^ TCM interventions can regulate the intestinal microbiota composition and improve the gut microecology, which may benefit the CNS disorders and enhance therapeutic outcomes.^132^ Patchouli alcohol (PA), a compound derived from the common traditional Chinese herb *Pogostemonis Herba*, stands as a principal bioactive constituent. Its application in murine models of AD ameliorates the ecological equilibrium of the intestinal milieu, concurrently repressing the activation of the C/EBPβ/AEP signaling pathway in the cerebral and colonic tissues.^133^ Consequently, PA efficaciously diminishes amyloid‐beta levels, impedes the aggregation of amyloid‐beta plaques, retards neuroinflammation within the cerebral regions of AD‐afflicted mice, and mitigates the hyperphosphorylation at various tau protein sites.^37^, ^133^ *Scutellaria baicalensis* root,^134^ *Morinda officinali* ^135^, ^136^ and Cyanidin‐3‐O‐glucoside^137^ exhibit analogous regulatory mechanisms. Moreover, a multitude of empirical investigations have substantiated the extraordinary effectiveness of TCM formulations in addressing the intricacies of AD.^138^, ^139^ These formulations, through the modulation of the intricate ecosystem of the gastrointestinal microbiota, evince efficacy for therapeutic interventions in AD. For instance, Huanglian‐Jiedu Decoction augments SCFAs levels, mitigates central and peripheral lipid metabolism disorders and inflammation, diminishes amyloid‐beta deposition, and augments cognitive function.^140^ Furthermore, TCM formulations such as Liuwei‐Dihuang Decoction,^141^, ^142^ Chaihu‐Shugan‐San,^143^ Jia‐Jian‐Di‐Huang‐Yin‐Zi Decoction^144^ efficacy in modulating the gastrointestinal microbiota to ameliorate the therapeutic outcomes of AD. Although other natural compounds with potential therapeutic benefits and minimal side effects may exist, they remain undiscovered or unidentified.

### 5.4 Fecal microbiota transplantation

Fecal microbiota transplantation (FMT) is a technique that transfers gut microbiota from a healthy donor to a recipient with a disturbed gut microbiota, aiming to restore the ecological balance.^145^ FMT has been explored as a potential therapy for neurological disorders. Sun et al. showed that FMT from WT donors improved cognitive function and reduced Aβ plaque load and soluble Aβ40 and Aβ42 levels in APP/PS1 mice.^146^ FMT also increased the expression of synaptic plasticity‐related proteins and the levels of beneficial SCFAs, especially butyrate, in the gut. Similarly, Fujii et al. reported that WT mice receiving FMT from human donors with AD, especially at a younger age, exhibited cognitive impairments compared with those receiving FMT from healthy donors.^147^

## 6 CONCLUSIONS AND FUTURE PERSPECTIVES

The correlation betwixt the enteric microbiota and AD has surfaced as a novel perspective in the amelioration of this affliction. O'er the bygone two decades, considerable advancement hath been attained, elucidating the potential of the enteric microbiota as a target for AD therapy. Despite these strides, there is a requisite to elucidate the causative relationship betwixt the enteric microbiota and AD to formulate efficacious and safe therapeutic interventions.

In adjunct to the enteric microbiota, the oral microbiota also exerts a potential sway on the pathogenic mechanisms of AD. Alterations in the oral microbiota impact the pathogenesis of AD through sundry mechanisms, including the initiation or exacerbation of neuroinflammation via hematogenous dissemination.^148^ The oral microbiota communicates with the enteric microbiota through the oral–gut–brain axis, and the imbalance in the oral microbiota may disrupt the equilibrium of the enteric microbiota, thereby exacerbating the pathophysiology of AD. A more profound exploration of the interplay betwixt the oral microbiota and AD will contribute to the exploration of novel therapeutic and preventive approaches. Understanding molecular interactions and the potential to ameliorate the condition of AD patients by modulating the oral microbiota is of paramount importance.

In pondering the prospective alleviation of AD susceptibility through microbial therapy, due regard must be accorded to its efficacy within vulnerable cohorts. Diligent inquiries into the curative ramifications of microbiota intervention measures are imperative, encompassing synergistic impacts, considerations of sample magnitude, and investigations into the consequences of protracted interventions. Furthermore, circumspect evaluation is warranted for the repercussions of alternative microbiota‐targeted therapeutic modalities on pharmacological interventions. Notwithstanding the expansive potential of gut microbiome modulation, extant strategies for regulating the gut microbiota in the context of AD remain inadequately refined. A more encompassing research undertaking is requisite to ascertain optimal intervention methodologies and their enduring ramifications, thereby providing viable and secure pathways for the prevention and management of AD. Such endeavors will contribute to elucidating underlying mechanisms, advancing deeper theoretical frameworks for interventions involving the regulation of the gut microbiota or the utilization of bioactive constituents.

## AUTHOR CONTRIBUTIONS

JL and YW: writing – original draft. JL, XD, and YW: data collection and integration. BL, WC, NZ, and HZ: conceptualization. BL, XD, and NZ: supervision. BL and NZ: project administration. WC and HZ: funding acquisition. All authors contributed to the article and approved the submitted version.

## FUNDING INFORMATION

This work was financially supported by the Natural Science Foundation of Heilongjiang Province (no. LH2023H073).

## CONFLICT OF INTEREST STATEMENT

The authors declare that the research was conducted in the absence of any commercial or financial relationships that could be construed as a potential conflict of interest.

## Data Availability

Data sharing is not applicable to this article as no datasets were generated or analyzed during the current study.
